# The Therapeutical Use of Iodinized Milk

**Published:** 1878-08-20

**Authors:** 


### THE THERAPEUTICAL USE OF IODINIZED MILK.

---

Lazansky, assistant at the clinic of Professor Pick, in Prague, has recently experimented with the view of determining the value of employing nurse's milk, impregnated with iodine, as a remedy in syphilis and other diseases in which iodine is indicated. A case is reported (*Viertelj. f. Dermatol. u. Syph. I.*, 1878) in which the result was extremely satisfactory. A nursing woman, suffering from constitutional syphilis, whose five months old child was likewise syphilitic, was given half a gramme (7½ grs.) of iodide of potassium twice a day. No medicine was given to the child. On the day this medication was begun, chemical examination of the milk and urine of the mother showed the presence of iodine in both these secretions. On the following day, the presence of iodine was also demonstrated in the urine of the child, On interrupting the administration of the medicine, the iodine disappeared from the secretions to reappear when its use was resumed. The child thrived remarkably well, and the manifestations of the disease disappeared rapidly in it as well as in the mother. The latter complained of no symptoms of gastric derangement, and she gained rapidly in flesh and strength. The mammæ remained large and full of milk, and no diminution of the lacteal secretion could be noticed. The quality of the milk also remained good. The author thinks, therefore, that the assertion so frequently made by obstetricians and others, that iodide of potassium is an antigalactogogue, is by no means proven. He also thinks, with Bouley, that there is a new combination entered into between the iodine and certain constituents of milk, which renders the medicine more assimilable. He adds, that not only in syphilis, but in other diseases of children and of adults, where iodine is indicated, this method of administration may be of use. The milk of animals might be iodinized by adding iodine preparations to their food, as had been successfully done by Lewald, Piogey, and others.—*Va. Med. Monthly.*

